# Counseling students' experiences and interpretations of purpose development

**DOI:** 10.1016/j.heliyon.2023.e13760

**Published:** 2023-02-15

**Authors:** Gitima Sharma, Mariya Yukhymenko-Lescroart, Katherine Bernal-Arevalo

**Affiliations:** a Department of Counselor Education and Rehabilitation, California State University, Fresno, United States; b Department of Curriculum and Instruction, California State University, Fresno, United States

**Keywords:** Life purpose, Purpose development, Counseling students, Identity, Wellness

## Abstract

The aim of this study was to explore the lived experiences of counseling students regarding their development of sense of purpose in life and further seek their recommendations for fostering sense of purpose within educational settings. In this study, we have used pragmatism as our research paradigm and Interpretative Phenomenological Analysis (IPA) as our data analysis approach to gain deeper insights into the phenomenon of purpose development and further use the findings of this study to suggest specific purpose-strengthening educational approaches. Based on the interpretative phenomenological analysis, we identified five themes that revealed purpose development as a non-linear process that involves exploring, engaging with, reflecting upon, articulating, and actualizing one’s purpose, and is influenced by both internal and external factors. In light of these findings, we discussed implications for counselor education programs that aspire to cultivate counseling students' sense of purpose in life as an important dimension for their personal wellness, which research has shown could further promote their professional development and career success.

## 1 Introduction

Sense of purpose plays an important role in contributing to students' motivation [1], goal-clarity [2], degree-commitment [3], college retention [4], and well-being [5]. A recent qualitative study among counseling students showed that students who felt driven to actualize their professional aspirations based on a strong sense of purpose in life articulated a greater sense of wellbeing, self-awareness, motivation, and dedication to keep persevering on their career path [6]. The focus of this study was to explore the lived experiences of counseling students regarding their purpose development to shed light on purpose-strengthening educational approaches.

There were several reasons for us to explore purpose development among counseling students. First, despite the significant role that life purpose plays, there is a lack of empirical understanding about how people awaken to, strengthen, and strive towards fulfillment of their purpose in life [1,2]. As a result, there is a lack of initiative in implementing counseling and educational interventions that could foster people’s sense of purpose. Moreover, the few studies that have been conducted on purpose development have primarily focused on high school and undergraduate students (e.g., Refs. [1,2,7]). To reduce this gap in literature, we conducted this study among graduate counseling students to shed light on the long-term pedagogical practices that could cultivate students' sense of purpose in life within educational settings.

Second, research has shown that counseling students often choose the counseling profession because of their own life experiences that shaped their sense of purpose in the direction of helping those who are undergoing similar hardships [6]. After beginning to encounter clients who share similar sufferings, counseling students often experience a growing sense of anxiety, depression, stress, and interpersonal difficulties [8]. During this time, if counseling students begin losing focus on their own well-being and personal development, it can lead to detrimental effects not only for their own health and success, but also for the wellbeing of their clients [9]. Supporting students to strengthen their sense of purpose could promote their personal well-being and ability to persevere [6].

Third, scholars have found that the professional success of counselors-in-training depends more on their individual characteristics, self-awareness, and personal development than on their school knowledge and theory (e.g. Refs. [10,11]). Experiencing a state of wellness is in fact critical for both “counselors' functionality and therapeutic effectiveness” [12], p. 3]. Professional organizations such as the Council for Accreditation of Counseling and Related Educational Programs (CACREP) and the American Counseling Association (ACA) have asserted that counselor education programs must support counseling students to develop strategies for self-care and wellness [[13], [14], [15]]. However, very few counseling programs incorporate initiatives to promote students' personal well-being [11,16,17]. Instead, the majority of counselor education programs focus on fostering counseling students' professional knowledge and external skills such as active listening through which they can benefit their clients [16]. Though important, these skills do not promise an effective counseling session in isolation from students' internal dispositions [11,16]. Through supporting students to experience wellness within the context of deepening their sense of purpose in life, counseling programs could help them experience optimum well-being and positive development, and thereby, fulfill the aim of the counseling profession to focus on prevention and on promotion of people’s positive functioning instead of only reduction of pathology [18].

## 2 Counseling students' wellness and life purpose

Becoming a counselor is a stressful journey that requires students to engage in consistent self-reflection and practice self-care to experience a deeper sense of well-being not only for themselves but also for others' well-being. Research has shown that counselors in training often undergo extreme stress and existential anxiety while trying to support clients who are facing various challenges [8]. In fact, a significant number of counseling students' score at clinical levels of psychological distress [8,12].

Empirical studies have highlighted the importance of personal growth, ego development, and self-awareness in supporting counseling students to overcome their own psychological distress and to further promote others' wellness [8,12,19]. Personal wellness and self-awareness also strengthen counseling students' self-esteem and professional identity [11,20]. Several scholars have therefore advocated for supporting counseling students to reflect upon their own values and beliefs, achieve positive growth, understand their inner self (thoughts and emotions) eloquently, learn the impact that their behavior has on others, and model self-care (e.g., Refs. [8,12,19,21]). However, wellness is often not formally addressed until the absence of focusing on counseling students' personal well-being is apparent, resulting in decline in students' professional competency and remediation [22].

Further, even if integration of wellness in counselor education programs has been increasing, there is a lack of clarity regarding the various dimensions and elements of wellness that are important for counselors training [17]. One of the important dimensions of wellness that counselor educators and scholars have emphasized upon is sense of purpose in life [6,23]. There are several counseling models and paradigms that have also conceptualized wellness in the context of life’s purpose. For example, Myers et al.’s [24] foundational theory on the wheel of wellness, referred to spirituality that encompasses sense of purpose as being the center of the wheel. Hattie et al. [25] further emphasized that individuals who lack self-direction and purpose in life are less likely to have an essential sense of wellbeing and motivation for self-care; thereby putting them at risk for both mental and physical illness.

According to a pioneer in the scholarship on counseling and wellness [26], maintaining a purposeful direction in life is the key to actualize a state of optimal wellness. Life purpose empowers people to manifest their highest potential towards the pursuit of their life’s most important goals [23,27]. Among counseling students, having a sense of purpose in life strengthens their desire to further contribute to their clients' well-being while role-modeling a life of holistic wellness and positive growth themselves [6].

Hence, to promote counseling students' personal wellness, counselor educators could focus on strengthening students' sense of purpose in life. One of the first steps in being able to do so is to gain a deeper understanding about the experiences and processes that influence development of purpose among counseling students. It is further important to note that a standardized approach towards promoting wellness among counseling students who represent diverse cultural values and identities would not be suitable given the close relationship between students' culture and well-being. Therefore, counselor educators and scholars must make an effort to explore how students from diverse cultural backgrounds perceive and cultivate different dimensions of wellness. This study represents interpretations and experiences of an ethnically diverse group of counseling students regarding actualizing personal growth and well-being within the context of their life’s purpose.

## 3 Review of literature on college students' purpose development

Purpose development is both an internally and socially constructed phenomenon [28]. Qualitative research has shown that purpose development is a non-linear process during which people lose and gain momentum, influenced by life transitions, identity formation, and external influences [7]. These influences include positive encouragement, mentoring, and experiential activities that college students encounter in educational settings, along with students' family history, cultural background, and spiritual beliefs [1]. For instance, in a phenomenological study [1], student-participants from minority ethnic backgrounds shared that their identity as students from minority and under-represented backgrounds inspired them to pursue their higher education to inspire other youth from similar backgrounds. Further, participants' families' immigration struggles shaped their purpose in the context of repaying gratitude to their parents through fulfilling their collective goals, such as securing a strong financial foundation for future generations.

Another qualitative study explored the phases that characterize purpose development among college students with disabilities [2]. Though not generalizable to all college students, this study illuminated some key phases of developing purpose: imagination, exploration, and integration [2]. The imagination phase involves students imagining and talking about their interests, skills, passions, values, and life goals. The exploration phase involves students taking active steps to explore their specific interests, skills, and goals. The integration phase involves students integrating their interests, perspectives, passions, activities, and goals into their evolving narrative of self and into their central purpose in life. This study further highlighted the various aspects of students' multiple intersecting social identities, personalities, societal roles, interests, and life experiences that played an important role in purpose development. The extent to which these aspects of self and social identities influenced purpose development was determined by the social value placed upon them in particular societies [2]. Thereby, suggesting the importance of conducting more research on purpose development among students belonging to different career and social contexts.

Recently, Sharma and Yukhymenko-Lescroart [27] integrated current research to inform the key pedagogical processes and phases of purpose development, which include purpose exploration, engagement, reflection, articulation, and actualization. Here, the phase of exploration focuses on supporting students to explore their personal and socio-cultural identity since it often influences their life’s values and aspirations. The phase of engagement highlights the importance of actively engaging in altruistic and community-based activities that could advance their and others' well-being. The phase of reflection focuses on providing students a dedicated space and time to reflect upon who they are and what they aspire to use their life for – within the context of their identity and life’s aspirations. The phase of articulation encompasses providing students the opportunities to narrate or write about their life’s purpose, and further articulate the connections between their personal identity, career goals, and sense of purpose in life. Lastly, the phase of actualization focuses on promoting students' goal-clarity and inner well-being to help them continue to persevere on the path of their life’s purpose.

## 4 Purpose of the present study

The central purpose of this study was to explore the lived experiences of counseling students regarding the development of their sense of purpose in life. The second purpose that guided this study was to understand counseling students' perceptions, interpretations, and recommendations for fostering sense of purpose within educational settings and thereby, shed light on purpose-strengthening educational approaches. Based on the previous studies on role of life purpose in contributing to college students' personal well-being and persistence (e.g. Refs. [4,5]), and on the importance of focusing on counseling students' wellness and personal growth [11], we believe this study can contribute to the literature on purpose development and counselor education.

## 5 Methodology

In this study, we have used pragmatism as our research paradigm and Interpretative Phenomenological Analysis (IPA) as our data analysis. Within pragmatic inquiry, the decision on the appropriate method for data analysis rests upon the nature of the research question and the method that might be most suitable to meet research goals [29]. Being interested in gaining insights into students' individual and collective experiences around purpose development led us to use the phenomenological approach of IPA as the data analytical method. Further, in line with pragmatic research paradigm that promotes action with practical utility [30], we discussed these experiences within the context of purpose-strengthening educational approaches.

### 5.1 Context and participants

The participants included 27 counseling students from a Hispanic-Serving and Asian American and Native American Pacific Islander-Serving Institution. The students belonged to the following counseling program specializations: Marriage, Family, and Children Counseling (MFCC) (30%), School Counseling (41%), Rehabilitation and Mental Health Counseling (18%), and Student Affairs and College Counseling (SACC) (11%). Their ages ranged from 23 to 47 years. Participant gender was 78% identified as female, and 22% identified as male. The majority (51%) of the students identified themselves as Latinx, 30% as Caucasian, 8% as more than one ethnicity or race, 7% as Hmong, and 4% as Filipino (see Table 1).

**Table 1:** Participants demographic information.

Pseudonym	Age	Sex	Race/Ethnicity	Program Emphasis
Mike	26	M	Latino	School Counseling
Stephanie	28	F	Caucasian	MFCC
Xavier	33	M	Latino	School Counseling
Stefan	39	M	Latino	MFCC
Viola	27	F	Caucasian	MFCC
Marie	25	F	Caucasian	School Counseling
May	24	F	Filipino	School Counseling
Adamaris	23	F	Latina	Rehabilitation
Delilah	46	F	Caucasian	MFCC
Amita	25	F	Indian/Greek	Rehabilitation
Mateo	25	M	Latino	Rehabilitation
Abraham	26	M	Latino	School Counseling
Grace	36	F	Caucasian	Rehabilitation
Angela	25	F	Caucasian/Latina	MFCC
Lili	25	F	Latina	MFCC
Lucilla	24	F	Latino	School Counseling
Hana	47	F	Caucasian	MFCC
Penelope	24	F	Latina	MFCC
Joanna	24	F	Irish	Rehabilitation
Eva	24	F	Latina	SACC
Jane	26	F	Latina	School Counseling
Molly	40	F	Latina	School Counseling
Amara	34	F	Latina	School Counseling
Kaj	25	F	Hmong	SACC
Houa	27	F	Hmong	SACC
Fabiola	29	F	Latina	School Counseling

### 5.2 Data collection procedure and participants sampling

Upon receiving approval from the University’s Committee for the Protection of Human Subjects, we recruited participants using purposeful sampling. The criteria for selection were that participants must be current graduate students pursuing their Master’s degree in a counseling-related specialization. At the initial stage of recruitment, the first author emailed all students who were enrolled in the counseling programs about this research study, and the third author, who is not an educator within the counseling program, presented the purpose of this study along with its ethical considerations during class sessions that were taught to students from all counseling specializations. After reading the purpose of this study and the informed consent, 27 students voluntarily participated.

The informed consent included details on participants' rights such as their right to withdraw from this study or to choose to not answer a question at any time without explanation and without penalty. The informed consent also mentioned that the interviews would be recorded, transcribed verbatim, and stored electronically in a password protected file only accessible by the investigators. For the protection of participants' identities, they were told that during the transcription process they will be assigned a pseudonym to ensure their anonymity and no other identifiable information will be collected or reported. After participants signed the informed consent, the third author scheduled and conducted semi-structured and purposeful interviews in a confidential setting. Each interview lasted for about 60 min.

### 5.3 Interview protocol

In accord with IPA, we created open-ended questions that were “*neutral rather than value-laden or leading*” [31], p. 63]. We specifically used the following interview protocol: (a) Please share about your sense of purpose in life and the process through which you awakened to your life’s purpose, (b) What experiences and factors influenced the development of your purpose? (c) How has the nature of your purpose transformed or strengthened overtime? (d) In your opinion, what are some of the factors that contribute to and/or hinder college students' purpose development? (e) Can you please share some suggestions and recommendations on how counselors and educators could strengthen students' sense of purpose?

### 5.4 Data analysis

To explore how participants process, interpret, and understand their own experiences in relation to purpose development, we followed Smith et al.’s [32] step-by-step process to analyze all 27 transcripts. For the first step, we read each of the transcripts multiple times to immerse ourselves within the data. We used the left margin on the transcripts to annotate the significant content and meanings that emerged from reading the participant’s lived experiences. During this phase of annotation, we ensured that we all took some notes for each of the response units. Some annotations reflected our attempts to paraphrase what the participant was expressing and/or the meaning we made of the experiences they were sharing [31].

As we read and re-read all participants' transcripts, we started noting the similarities and differences that were emerging across the narratives. We then began the process of initial noting, during which we examined the specific ways in which participants talked about, understood, and thought about purpose development. As a result, we developed emerging themes. We ensured that these themes represented a higher level of abstraction that could allow theoretical understanding, but were also grounded in participants' particular narratives and direct quotes [31]. This process of writing initial themes helped us to reduce transcript detail volume while maintaining the complexity and mapping the patterns between notes. While writing initial notes we strove to bracket the ideas gained from analyzing one transcript before moving on to the next participant’s account, repeating the analysis process until we finished the review of all 27 transcripts.

After taking initial notes for each of the transcripts, we wrote the emergent themes on a separate sheet of paper. Doing so helped us search for connections across the emerging themes and chart how the themes fit together throughout the transcripts. We found that some of the themes clustered together as one theme, and others were important insights but did not represent significantly meaningful concepts. During this process, several themes were dropped while others were highlighted as either most recurring themes or unique findings. At this stage, we also identified clusters of themes and labeled these clusters.

Next, we looked for patterns across the cases to categorize the connections among all transcripts and highlight the most potent themes. We then created a table of superordinate themes that highlighted the richness of data instead of frequency of certain findings. Finally, we wrote our initial draft on findings to represent the meanings inherent in participants' lived experiences in relation to purpose development – especially within the educational settings.

### 5.5 Establishing trustworthiness and credibility

To establish trustworthiness and credibility, we strove to address the critical threat of researcher subjectivity in qualitative research [33] through reflecting upon our own positionalities, values, and experiences that could influence our interpretations of participants' responses. To further ensure objectivity, we engaged in investigator triangulation instead of one person being the sole analyst of data. Moreover, the third author, who is not a counselor educator and does not have a background in research on purpose, suggested initial themes, thus reducing risks of imposing our own standpoints and expectations on data.

Investigator triangulation was thus utilized through engaging multiple investigators to attend to this study and confirm its findings [34]. Specifically, after the third author had written the initial draft of findings, and all the co-researchers reviewed the transcripts and took initial notes for each participant, we met to share our varying perspectives on the data. Thereby, light was shed on the insights, themes, and knowledge gained from the data.

We further prepared detailed protocols and maintained a track record of the analysis process, including objectives, research design, and methods of the study. We maintained the idiographic nature of IPA by keeping all participants' accounts separated or bracketed to ensure the individuality of each emerging theme. We did so by following Smith et al.’s [32] recommendation on analyzing the narratives of each participant separately, bracketing the contents and ideas that emerged from each individual, and promoting an in-depth understanding of participants' experiences at the individual and collective levels.

As a final step, we engaged in member checking by sharing the transcripts and initial notes with the participants [33]. In this way, we attempted to triangulate our findings by comparing our findings as co-researchers and examining the data across multiple participants. To support the readers in determining the study’s transferability, we stated participants' direct quotes within the findings section.

## 6 Findings

The data analysis resulted in 14 sub themes and five overarching themes. Table 2 shows the individual components of each theme, which helped us to identify college students' experiences and interpretations around purpose development.

**Table 2:** Graduate counseling students' experiences of developing purpose.

Theme	Sub-themes	Descriptive Codes
Perception of Purpose	Describing their purpose	Helping others
		Making a difference
		Causing an impact
	The purpose of others	Everyone has a purpose
		Purpose is the same for everyone vs. Purpose is different for everyone
	Purpose as part of something bigger	The Universe
		God
		A calling
		“Meant to be”
	Purpose linked to career	Purpose associated with career choices
	Difficulty talking about purpose	Difficulty articulating thoughts
		Purpose rarely talked about/reflected on
Awakening to Purpose	Experiences	Life Experiences
		School Experiences
		Work Experiences
	The Self	Self-realization
		Self-reflection
		Self-care
		Self-awareness
Factors that Influence Purpose	Internal Influence	Fulfillment
		Spiritual and Religious Beliefs
		Following own will
	External Influence	Family influence
		Mentor influence
Transformation of Purpose	How Purpose Changes	Purpose can change with time vs.
		Purpose cannot change with time
	What Changes Purpose	Life experiences
		Family growth/change
Counselors' and Educators' Responsibility in Fostering Purpose	Awareness of Purpose	Counselors must be aware of their purpose
	Counseling Techniques	Building relationships
		Listening
		Being Present
		Encouragement/support
	Promoting Purpose in Educational Institutions	Classroom curriculum
		Career workshops
		Hands-on experience

### 6.1 The perception of purpose

All students believed that their life had a purpose, with most of them attributing their purpose to “helping others” and “making an impact,” indicating an altruistic sense of purpose centered upon serving others. This description was one with a selfless concern for assisting those in need. Furthermore, many students stated everybody had a purpose, but it can develop differently for everyone. Mike, a school counseling student, explained, “I believe we all have a sense of purpose in life. The key thing, though, is finding that purpose and being in tune with it.” Finding purpose was frequently described as a journey of discovery that each person experienced uniquely.

Students' views diverged when they discussed whether students had one unifying purpose or different purposes. Very few students described purpose as the “ultimate,” relaying that everybody was bound by one purpose. Conversely, a few shared that purpose could differ for everyone depending on their unique set of choices, skills, and experiences. Lucilla, a first-year graduate school counseling student, explained, “I don’t know if we all have a ‘meant to be’ purpose, but I do believe that we can all find a purpose based on our gifts, talents, and life experiences.” Despite the considerable awareness that counseling students had about their purpose, many students had trouble articulating their thoughts and discussing them. Some students admitted that they rarely talked about purpose or that it was their first time thinking about it. As Amita divulged, “no one has asked about [my purpose] until now, which is probably why I’m struggling with these questions a little.” A few more students stumbled over their words and recognized that talking about their purpose was a rare opportunity.

### 6.2 Awakening to purpose

Awakening to purpose is the process by which purpose becomes clear to an individual. Several students reported that their awakening was related to one or a combination of life events, including their work and education experiences. These students recalled life experiences such as receiving a diagnosis, having a child, losing someone tragically, or converting to a religion that helped them awaken to their purpose. For some students, the process was gradual, with a culmination of experiences leading to their awakening. Hana explained, “I believe that all the struggles I went through as a kid, being in an abusive marriage, being a single mom, are all factors.” Several students highlighted how the challenges in their life, such as isolation, bullying, abuse, and loneliness, ignited the desire to help others who might experience similar situations as them in the future. A few students shared the disappointment that emerged from their experiences in school settings helped them awaken to their purpose. For example, Stephanie shared an interaction they had experienced with their counselor:

Let’s not focus on college because it is not an option for [student]. Instead, let’s focus on her actually graduating high school.” Hearing those words come from my high school counselor made me realize how angry it made me that someone would allow my learning disability to define and determine my future. That was the main moment that I realized that no individual should have a limit to where they could go in life.

Students also referred to awakening to their purpose through the positive relationships with their teachers or counselors and work experiences (e.g., jobs, volunteer work, and internships). Some students articulated that their hands-on work experiences led to their purpose. Some experiences revealed that what they had initially chosen was not the right fit and inspired them to pursue more meaningful careers. For others, work experiences were valuable, allowing them first to try out and realize what they wanted to do, or to affirm that they had chosen the correct path. As Eva articulated:

I was working for a nonprofit, and I did a lot of outreach in our presentations, primarily with the Hispanic community. I came across a lot of undocumented students who just did not know at all that they could go to college, and it just kind of really made me realize the need that is out there for high school counselors.

Additionally, many students emphasized the importance of self-awareness as a form of awakening to one’s identity, which is imperative for understanding one’s purpose. Students shared that in the process of their identity development, reflecting upon their purpose was critical. For example, Molly explained that the ability to “know oneself” comes from reflections and self-realization, which is critical for understanding one’s purpose in life. Furthermore, self-awareness played an important role in enabling students to “tune in” with who they were and for what purpose they aspired to use their life. In this case, developing self-identity became one of the most significant aspects, not only for their sense of purpose to emerge but also for further strengthening it. In essence, students emphasized upon the role of exploring who they were; actively engaging with their interests, passions, and causes they cared about; reflecting upon their purpose in life; articulating their purpose; and making efforts to live “in tune” and actualize their purpose as some of the phases of purpose development.

### 6.3 Factors that influence purpose

The factors that influenced purpose were divided into two groups: internal and external. Internal influences came from within; for example, personal thoughts, feelings, beliefs, attitudes, and self-concepts. Conversely, external influences were the outside forces that had an impact on students' decisions and thoughts. The majority of students stated that their purpose was internally driven and strengthened by feelings of contentment, passion, satisfaction, and fulfillment that they experienced while walking on the path of their purpose. For some students, a sense of purpose stemmed from their religious identity or spirituality. Students explained how being part of a religion, following religious beliefs, or merely being in touch with their spirituality influenced how they developed their purpose. Spiritual and religious beliefs were frequently associated with feeling driven to serve God, humanity, and the universe.

Additionally, several students also talked about the external factors that influenced their sense of purpose. For example, some students shared that their family members inspired them to believe in themselves, encouraged them to pursue their purpose, and provided them the practical support needed to fulfill their goals. Similarly, students reported the value of advice, resources, and guidance they received from their faculty advisors, mentors, counselors, youth leaders, and teachers. They especially emphasized the positive role of encouragement that cultivated in them the desire and confidence to pursue their purpose. Furthermore, with mentors guiding them along the way, they could develop their interests and skills, further leading towards their life’s purpose.

#### 6.3.1 Transformation of purpose

All students' narratives highlighted their perceptions about whether sense of purpose remains stable or changes over-time. Specifically, some students believed that their overall purpose would not change over-time. For example, a student who mentioned that their purpose was to help others believed it was the foundation of their purpose and would not transform. Xavier stated, “I chose this career just to help people. I know with work and family changes … I still have to do what I’m destined to do.” Students who believed that their purpose would not change perceived time as a catalyst to gain the knowledge, skills, and experiences they needed to clarify and further strengthen their purpose.

On the other hand, some students believed that life’s purpose was ever-changing. These students perceived major life events, changes in personal beliefs and perspectives, and experiences yet to come as factors that could transform their purpose. A few students attributed family growth as a major reason for change in their purpose. For example, Jane said:

I know once I start working, I’ll have this drive to start helping students, and I am going to want to do something more. Also, when I have a family, then my purpose in life will be to be a great mother. So, it is always changing with any new life event.

The students who shared that their purpose might change with time also spoke about how their purpose was currently thought about in terms of their careers, but that time could change their priorities and hence their purpose.

### 6.4 Counselors' and educators' responsibility in fostering purpose

All students agreed that counselors were in part responsible for helping their clients in cultivating their purpose. Students believed that counselors could foster purpose by practicing various counseling techniques such as listening without judgment, being present, providing encouragement, showing support, and building positive relationships. Some students also emphasized the importance of counselors being aware of their purpose, explaining that one cannot help others without helping oneself.

Furthermore, some students mentioned the role that educational institutions could play in developing purpose among students. Lili asserted, “Exposing students to [purpose] as much as possible is critical. It will help students develop a sense of themselves, as well as a sense of purpose.” Through implementing classroom curriculum, individual and group counseling, schools could ensure that students would have conversations about purpose as they attain their education. The exposure to these purpose-oriented conversations at school could facilitate students to have self-realization and self-reflection, leading to the awakening of purpose. Furthermore, students stressed that students often have difficulty awakening to their purpose and that implementing mentoring programs in school could guide many students who felt lost.

Additionally, many students believed that students' purpose was closely related to their career. They explained that counselors could simplify the challenging experience students faced when choosing a career through purpose-centered career counseling to help students explore their career interests within the context of their life’s purpose. Counselors could also provide students with information about various career options and opportunities that might align with their purpose. Students declared that an effective way to do this was by coordinating events and workshops focusing on connecting students with internships and community-based organizations that aligned with their life’s purpose.

## 7 Discussion

Professional counseling organizations have asserted the importance of supporting counselors-in-training to promote their clients' well-being through modeling wellness and consistent efforts for self-care and positive development (e.g. Refs. [13,15]). Several scholars in counselor education (e.g. Refs. [8,11,12,17,19]) have also advocated for exploring and integrating different dimensions of wellness within counselor education curricula to support counseling students' personal well-being. Through introducing, reinforcing, and helping counseling students to practice wellness-oriented practices, counselor educators could prevent students' burnout and further help them to promote their clients' well-being based on preventive and holistic approaches that characterizes counseling profession [17].

One of the important dimensions of wellness is purpose in life. The aim of this study was to explore the experiences and interpretations of purpose development among counseling students and further use the findings of the study to shed light on purpose-strengthening educational approaches. The findings showed that all students who participated in this study, articulated their life’s purpose as advancing a positive change in society by helping, serving, and guiding other people. These students emphasized that the key to living a fulfilling life that is undefeated by current hardships is to live in “tune with” their life’s purpose. However, students shared that even if they realize the value of having a sense of purpose in life and felt clear about their life’s purpose, they struggled with strengthening, expressing, and fulfilling their purpose. Students also added that despite the important role that purpose played in their life, taking part in this study was their first time discussing and articulating their life’s purpose, which they found very meaningful. Students, therefore, suggested creating more educational opportunities that could help them to reflect upon, strengthen, and actualize their purpose in life especially in relation to their own wellness- and professional development-oriented endeavors.

The students' narratives helped in gaining insights into the specific factors and processes that influence their purpose development, and hence shed light on the purpose-strengthening pedagogical approaches. Students' experiences highlighted that the process of purpose development was fluid in nature and involved specific phases. In line with Vaccaro et al.’s [2] theory, the present study showed that the development of purpose is a journey that usually began with students actively exploring who they are in the context of their socio-cultural backgrounds, life experiences, interests, abilities, passions, and goals. Apart from exploring and deepening their sense of identity, students highlighted the importance of actively engaging with the causes they cared about. The process of purpose development also included consistent time periods of reflecting upon and articulating their life’s purpose. Students further emphasized that awakening to, transforming, and fulfilling their purpose was a life-long phenomenon that required them to persevere on the path of various goals that resonated with their life’s purpose.

In addition to illustrating the process of awakening to and further strengthening their sense of purpose in life, the students highlighted several internal and external factors that influenced their purpose development. Students specifically shared personal beliefs, attitudes, skills, interests, and values as internal factors that influenced their life’s purpose. Students further explained work experiences, family support, spiritual guidance, mentorship, and encouragement from faculty advisors, counselors, youth leaders, and teachers as the external factors that helped in fostering their sense of purpose.

Students' narratives also highlighted the importance of seeing purpose development as a lifelong phenomenon that can go through significant moments of transformation based on changing priorities and self-realizations. This finding reaffirmed scholars (e.g., Refs. [7,28]) assertions that students' purpose development is influenced by various life events and transitions such as expanding family, changing career, falling ill, losing a loved one, going through trauma, and changing religion. Students' stories further showed that the nature of people’s purpose is often related to the personal hardships and adversity they themselves have overcome. In relation to this finding, a Buddhist philosopher and peacebuilder [35] expressed:

By triumphing over great poverty, a person who has been poor can give hope to others who are struggling with financial hardship. By regaining vitality and good health, someone who has been battling illness can light a flame of courage in the hearts of those in similar straits … By extension, the personal problems we face can serve as sources of tremendous encouragement to others who are suffering from similar problems, spurring them to do their best and remain undefeated (p. 85).

### 7.1 Implications for counselor education

This study provides several implications for counselor educators considering cultivating students' sense of purpose in life as an important dimension for their personal wellness. The findings of this study showed that all students' journeys of purpose development and the extent to which they feel clear and committed to their purpose are different. To help counseling students who might be in the phase of either exploring, engaging with, reflecting upon, articulating, and/or actualizing their purpose in life, counselor educators can either enrich their existing courses with purpose-centered pedagogical processes or develop new courses focused upon students' self-exploration, career development, and wellness in the context of their sense of purpose in life. In such courses, counselor educators can incorporate self-exploration activities using tools such as values-clarification surveys that can help students reflect upon life’s purpose.

Counselor educators can also include reflective assignments that can promote students' understanding of community needs and how they can use their life and counseling profession to actualize their vision for themselves and the world. Given the important role of students' cultural backgrounds and previous experiences in shaping their purpose, it is important for counselor educators not to impose a certain form of purpose on students or expect that all students' purpose will be linked with the counseling profession. This study further highlighted that both internal factors such as personal values and external factors including cultural background play an important role in purpose development. Thus, counselor educators are encouraged to create spaces for open dialogues on how students' socio-cultural experiences and intersecting identities have shaped their purpose. Within this context, counselor educators can also discuss the specific populations that students as counselors-in-training would like to work with. Counselor educators could further include reflective assignments on how students' personal life experiences and socio-cultural identity intersect to shape their life’s purpose – within courses that focus on multicultural issues of counseling.

Based on this study’s findings, it is important for educators, counselors, and advisors to understand the unique phase of purpose development with which a student might be struggling and for which might be seeking resources. For example, some students might be in the phase of purpose exploration and would benefit from tools that can help them clarify their life’s purpose. Other students, such as most of the graduate counseling students in this study, might be clear about their purpose but might be struggling with lack of opportunities to express their purpose. These students can benefit from career- and wellness-oriented workshops that can help them to set short-term and long-term goals that might resonate with their purpose and further connect them with organizations that might align with their purpose. Since all students' journeys of purpose development are unique and require personal space and time, counselor educators can leverage technology to incorporate asynchronous learning experiences that can support students to reflect upon and deepen their sense of purpose at their own pace.

Students in this study also shared that many times even if they are aware of their purpose in life, they do not feel confident about fulfilling their life’s purpose. Students emphasized the important role of encountering faculty advisors who believed in them, cared for their future aspirations, and provided them the resources needed to fulfill their aspirations. Hence, counselor educators' own humanity rooted in the belief that every student’s life has a purpose that they can fulfill [36] is critical for supporting students to pursue a life of purpose. Rooted in such a belief, counselor educators can actively communicate faith in students' future possibilities and life’s purpose to help them persevere.

Lastly, given the overwhelming nature of counseling programs and the amount of time it can take to directly apply counseling knowledge and skills to fulfill one’s purpose, it is important to instill hope and courage in students' hearts. In addition to counselor educators, students' mentors, advisors, supervisors, and counselors could use encouragement-based counseling skills such as actively communicating faith in students' inherent potential, enhancing students' awareness about their own unique strengths, and inspiring students to keep making efforts in the direction of actualizing their life’s purpose [37].

## 8 Limitations and future research directions

As discussed by Gobo [38], in qualitative studies, issues of sampling, representativeness, and generalizability ought to be faced in a practical approach through the lens of social significance. Because the goal of this qualitative study was not to generalize but rather to provide a contextualized understanding of the experiences and interpretations of purpose development among graduate students in counseling to discuss purpose-strengthening counseling and educational approaches, limitations related to generalizability regarding our sample need to be carefully considered. The two main and linked issues are related to the representativeness of the sample and the generalizability of findings. In this study, we sampled graduate students in various counseling programs to know their experiences related to purpose development and interpretations. We interviewed students pursuing graduate degrees in counseling who, given the service-focused nature of the programs, were relatively homogeneous in terms of awareness and nature of their life purpose. While our sample might not represent all students with all the variability of their experiences related to purpose development, these students may graduate and new students may enroll, but the nature of the purpose development and interpretation may not change much. Certain findings such as the close overlap between students' career choice and life’s purpose might be limited to this group of students. For instance, the students in this study described their purpose as helping specific groups of people whom they will serve as counselors. However, students from other career paths might not speak of the closeness between their life’s purpose and career aspirations in a similar manner. Therefore, additional studies are needed to examine whether the findings from this study transfer across other career specializations. Future studies can also explore how purpose continues to develop at different points of people’s lives, for example, a college student compared to someone at the height of their career.

Another limitation of this study is its focus on only one dimension of wellness, which is a sense of purpose in life. Given the rationale for making greater efforts to promote counseling students' personal wellness and self-awareness, future studies are needed to assess how counseling students develop other aspects of wellness. Lastly, the generalizability of findings needs to be considered within the context of conditions under which our findings were drawn, or the generalizability about the nature of a process. This study does not aim at giving a quantitative objective description of graduate students' purpose development, but can help in understanding some of its essential aspects, basic processes, crucial experiences, key factors which reflect how graduate counseling students experience and interpret purpose development and help us identify approaches of how purpose can be further encouraged and strengthened. Therefore, findings are not generalizable as descriptions of how purpose is strengthened in graduate students across various programs in general, but they are generalizable as descriptions of how purpose can be strengthened in students within similar contexts as participants in this study. The findings of this study can therefore be used to inform purpose-strengthening counseling and educational approaches among college students. Hence, another future research direction is to conduct more experimental studies that could implement and evaluate the effectiveness of specific purpose-centered interventions within the educational settings.

## 9 Conclusion

Given the importance of cultivating counseling students' well-being and internal dispositions in addition to their professional knowledge and skills [15], we conducted this study to explore counseling students' purpose development as an important wellness dimension. Through engaging in in-depth interviews, we found that purpose development is a non-linear process characterized by various phases, including exploring their self-identity; actively engaging with their interests, passions, and causes they care about; reflecting upon the impact of their life experiences in shaping their life’s purpose; finding opportunities to confidently articulate their purpose; and making efforts to live “in tune” with and actualize various goals that might resonate with their purpose. This study also highlighted several factors that influence purpose development. In the future, more studies are needed to explore the role of specific factors such as life’s challenges, cultural values, family’s history, and career experiences in shaping students' life purpose. Future research could also focus on implementing and evaluating the effectiveness of educational approaches that this study highlighted.

## Author contributions

Gitima Sharma: Conceived and designed the experiments; Performed the experiments; Analyzed and interpreted the data; Contributed reagents, materials, analysis tools or data; Wrote the paper.

Mariya Yukhymenko-Lescroart: Analyzed and interpreted the data; Contributed reagents, materials, analysis tools or data; Wrote the paper.

Katherine Bernal-Arevalo: Analyzed and interpreted the data; Wrote the paper.

## Funding statement

This research did not receive any specific grant from funding agencies in the public, commercial, or not-for-profit sectors.

## Data availability statement

The data that has been used is confidential.

## Declaration of interest’s statement

The authors declare that they have no known competing financial interests or personal relationships that could have appeared to influence the work reported in this paper.

## Ethics approval

All procedures performed in studies involving human participants were in accordance with the ethical standards of the institutional and/or national research committees.

## Consent

Informed consent was obtained from all individual participants included in the study.
